# Consensus Guidelines for Improving Quality of Assessment and Training for Neuromuscular Diseases

**DOI:** 10.3389/fgene.2021.735936

**Published:** 2021-11-10

**Authors:** Tina Duong, Kristin J. Krosschell, Meredith K. James, Leslie Nelson, Lindsay N. Alfano, Katy Eichinger, Elena Mazzone, Kristy Rose, Linda P. Lowes, Anna Mayhew, Julaine Florence, Wendy King, Claudia R. Senesac, Michelle Eagle

**Affiliations:** ^1^Department of Neurology, Stanford University, Palo Alto, CA, United States; ^2^Children’s National Hospital, Washington, DC, United States; ^3^Department of Physical Therapy and Human Movement Sciences, Northwestern University Feinberg School of Medicine, Chicago, IL, United States; ^4^The John Walton Muscular Dystrophy Research Centre, Newcastle Hospitals NHS Foundation Trust, Newcastle University, Newcastle upon Tyne, United Kingdom; ^5^Department of Physical Therapy, University of Texas Southwestern Medical Center, Dallas, TX, United States; ^6^Department of Pediatrics, The Ohio State University, Columbus, OH, United States; ^7^Abigail Wexner Research Institute at Nationwide Children’s Hospital, Columbus, OH, United States; ^8^Department of Neurology, University of Rochester Medical Center, Rochester, NY, United States; ^9^Department of Child Neurology, Catholic University Policlinico Gemelli, Rome, Italy; ^10^Discipline of Physiotherapy, Faculty of Medicine and Health, The Sydney Children’s Hospital Network, University of Sydney, Sydney, NSW, Australia; ^11^Department of Neurology, Washington University School of Medicine, St. Louis, MO, United States; ^12^Department of Neurology, The Ohio State University, Columbus, OH, United States; ^13^Department of Physical Therapy, University of Florida, Gainesville, FL, United States; ^14^ATOM International Ltd., Newcastle upon Tyne, United Kingdom

**Keywords:** clinical outcomes assessment, clinical evaluation education, neuromuscular disease (NMD), evaluator training, clinical trial readiness

## Abstract

Critical components of successful evaluation of clinical outcome assessments (COAs) in multisite clinical trials and clinical practice are standardized training, administration, and documented reliability of scoring. Experiences of evaluators, alongside patient differences from regional standards of care, may contribute to heterogeneity in clinical center’s expertise. Achieving low variability and high reliability of COA is fundamental to clinical research and to give confidence in our ability to draw rational, interpretable conclusions from the data collected. The objective of this manuscript is to provide a framework to guide the learning process for COAs for use in clinics and clinical trials to maximize reliability and validity of COAs in neuromuscular disease (NMD). This is a consensus-based guideline with contributions from fourteen leading experts in clinical outcomes and the field of clinical outcome training in NMD. This framework should guide reliable and valid assessments in NMD specialty clinics and clinical trials. This consensus aims to expedite study start up with a progressive training pathway ranging from research naïve to highly experienced clinical evaluators. This document includes recommendations for education guidelines and roles and responsibilities of key stakeholders in COA assessment and implementation to ensure quality and consistency of outcome administration across different settings.

## Introduction

**FIGURE 1:**
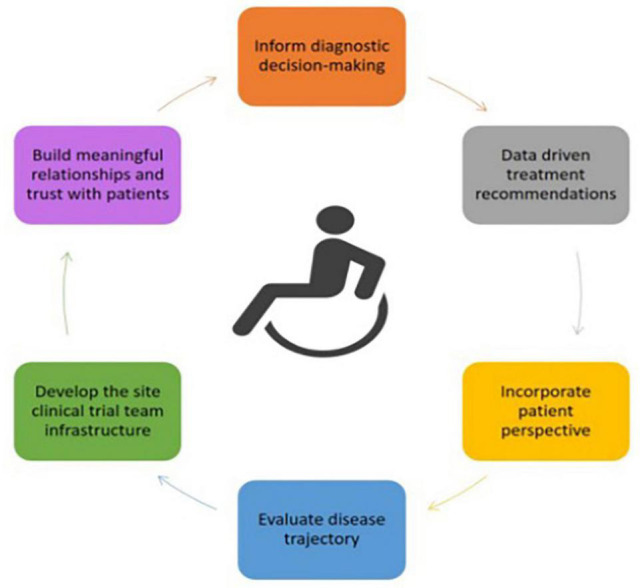
Purpose of high quality Clinical outcome assessment (COA).

Neuromuscular disorders (NMDs) are inherited or acquired conditions resulting in progressive muscle weakness, fatigue and loss in function. Disease onset varies from being present at birth to emerging in adulthood with a wide range of maximal function achieved and variability in trajectory of progression. Clinical outcome assessments (COAs) are used to document the natural history of the disease, evaluate the effectiveness of various therapies, support the registration of investigational drugs, and monitor the impact of therapies over time. It is therefore crucial that COAs are administered and scored in a reliable and valid manner to provide insight on the impact of NMD processes and progression by tracking clinical changes that may play a role in clinical decision making and trial design (Figure 1).

Methodologic variables that have possibly contributed to failed trials include lack of, rushed, or poorly designed clinical evaluator training and competency (Kobak et al., 2004). Variables that contribute to accurate COA administration go beyond clinical experience, including conceptual understanding of the scale and disease, didactic training on the study protocol/objectives, ability to interact and motivate patients across the lifespan, applied learning, and frequency of education to reduce post-training evaluator drift. Drift is defined as decreased consistency of rater functioning over time (Congdon, 2000; Wolfe et al., 2001; Mulsant et al., 2002; Kobak et al., 2004, 2006; Jeglic et al., 2007). Drift occurs for many reasons such as lack of familiarity with the scale or scoring, inadequate training, decreased attention/fatigue, personal interpretation of the scoring system, and loss of content knowledge for decision making over time (Congdon, 2000; Wolfe et al., 2001).

Validity and interpretation of clinical findings can be significantly compromised with poor reliability of COAs. Establishing both intra-rater and inter-rater reliability is essential to ensure that any changes in a patient’s performance are due to the disease progression rather than evaluator error. Therefore, to ensure assessments are valid and reliable within site clinical evaluators (CE) for clarity multi-site trials or within repeated clinical visits for the same person, we propose a guideline for optimal data collection through standardized training.

Despite the importance of training CEs to perform COAs, there are few published standards to guide the selection and preparation of CEs to administer COAs in clinical trials (Mulsant et al., 2002). This leads to ambiguity in training methodology that may have a significant impact on the success or failure of trials due to variability and inconsistency in data collection (Kobak et al., 2004; Targum, 2006). In 1978, the Muscular Dystrophy Association (MDA) supported the very first studies documenting the natural history of Duchenne muscular dystrophy (DMD) (Brooke et al., 1981). There were key learnings for the crucial need to appropriately educate CEs in the reliable and valid administration of COAs. First, there needs to be established, validated and reliable outcome measures that have the power to prove a positive or negative effect in the disease (Brooke et al., 1983). Second, to ensure reliable administration, significant investment of time and money is essential for ongoing and regular quality training, even for those experienced clinical evaluators.

Many NMDs are progressive in nature, requiring trials to be performed longitudinally. For longitudinal, multi-site studies a comprehensive training program including didactic and applied education improves reliability of COAs (Gibbon et al., 1981; Kobak et al., 2004; Targum, 2006; Walker et al., 2014). Studies that limited training to one occasion, at an initial investigator meeting, resulted in poor reliability in administration of COAs (Demitrack et al., 1998).

International studies pose unique challenges with diverse cultural and language needs, as well as varied educational and experiential backgrounds that may impact accurate administration of COAs (Jeglic et al., 2007). Although documented inter- and intra-rater reliability could increase confidence in the robustness of data, only a few studies have used methods to document competencies in performance of COAs such as reliability testing and/or procedural knowledge through quizzes or skills demonstration (Personius et al., 1994; Escolar et al., 2001; Targum, 2006; Mayhew et al., 2007; Walker et al., 2014; Glanzman et al., 2018; Krosschell et al., 2018).

In this article, we describe two essential training components: 1. Didactic training to ensure a strong foundation of knowledge of the disease process and COA procedures, 2. Applied or practice-based training (Kobak et al., 2004, 2005; Targum, 2006; Jeglic et al., 2007; Steeves et al., 2007). One reason for the lack of adoption of universal education and training guidelines could be that the process of training CEs can be costly and time-consuming; and if not initiated early enough may lead to study start-up delays.

This guide aims to provide a framework for an education and skill acquisition training plan for the administration of COAs. The plan may be used both within and across sites to assess the impact the NMD has on an individual’s strength, function, and participation in daily activities. The primary objective is to describe a common framework for improving standardization and accuracy in the education and administration of COAs in NMD. These recommendations are intended to provide a construct for clinicians, investigators, industry, pharmaceutical companies, and clinical research organizations to develop their own education and training plan that encompass key components required for quality administration of COAs. We hope these guidelines developed by a group of leading expert trainers in NMD with a wide breadth of experience from early natural history studies to international multi-site clinical trials will provide a pathway to improve COA implementation across different types of studies and programs involving NMD. This framework will promote efficient, reliable, valid administration and scoring of COAs administered by CEs.

## Materials and Methods

An international group of 14 physical therapist (PT) clinical researchers with expertise in NMD disease and COAs attended a 1 day in-person meeting in Warrenton, VA, United States on December 04, 2015, to develop guidelines in COA administration in Duchenne Muscular Dystrophy (Supplementary Material). Since that time, there has been a dramatic increase in clinical trials for rare NMDs and we decided to have 3 subsequent virtual meetings to evaluate current practices and propose guidelines from experience and lessons learned for CE qualifications and trainings from these studies. Proposed guidelines included minimum training criteria and recommendations for implementation.

The contributing clinical experts have on average 29.1 years of clinical experience and 18.0 years of training of COAs in NMD (Table 1). These PTs have been leaders in establishing the current standards and recommendations used in multi-site studies and clinical trials for drug approvals through the Food and Drug Administration (FDA) and European Medicines Agency (EMA) over the last 40 years.

**TABLE 1:** Clinical outcome assessment (COA) physical therapists experience.

**COA Expert**	**Clinical Experience (years)**	**Research Training Experience (years)**	**Neuromuscular Experience (years)**
1	19	15	17
2	42	22	15
3	18	10	17
4	25	8	14
5	40	35	20
6	13	7	11
7	34	10	15
8	43	39	39
9	43	43	43
10	39	15	30
11	20	11	18
12	30	16	16
13	21	11	16
14	21	10	18
**TOTAL**	408	252	289
**Min**	13	7	11
**Max**	43	43	43
**Average**	29.1	18.0	20.6

### Recommendations for Clinical Outcome Assessment Education

**FIGURE 2:**
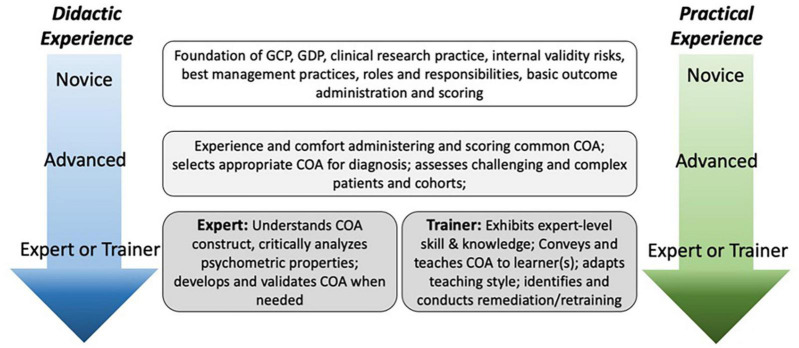
Levels of COA experience and knowledge.

Because experience levels among CEs can be diverse, we have provided guidelines for both *novice* and *proficient* evaluators (Figure 2). We deem a novice evaluator to have minimal clinical experience and/or, research experience and/or proficiency administering the COAs. Proficient evaluators have experience with the NMD patient population and have had prior training on the COAs. These groups require different teaching and training approaches. It is important to ensure novice and proficient learners start their training with a similar foundation of knowledge whilst still recognizing and acknowledging a CEs experience. With increased clinical and research experience, novice learners may eventually become proficient learners requiring different approaches to move beyond basic administration of the COA to active learning and critical thinking indicating integration of basic knowledge, understanding and interpretation of the content based on the patient population (Noonan et al., 2012). This is important for clinical trials that may have an impact on the historical phenotype of the population requiring the CE to analyze movement patterns and score the COA in a standard manner with a phenotype that may have not been previously taught or described. The level of training that will be required of a CE will depend on whether the COAs will be implemented in a clinic setting or in a clinical trial.

#### Training Process

This consensus guideline provides practical suggestions to ensure objective and accurate administration of COAs. Key stakeholders should be identified prior to study initiation to develop a training plan which should include:

✓ Qualification requirements of CEs.
✓ Contents of training.
✓ Testing methods and monitoring to ensure competency of accurate COA administration and scoring across a trial to minimize drift.

The training plan should include educational material that addresses different types of learners. Additionally, the training plan should also include an escalation plan to upskill CEs who do not meet professional and experiential criteria of training at the time of study start up, as this may impact the data collection.

The training plan should also address a strategy regarding CEs who have been trained and do not evaluate a patient. An example would be a novice CE who has been trained for 3 months and has not assessed a patient within the clinical trial. This affects information retention and increases the risk of drift and associated variability in COA administration. Methods to mitigate this may include a re-training plan for those CEs in the instance of delayed study start up or patient recruitment at sites.

#### Training Methods

Basic training of COAs should not only include information about scale administration and item-by-item scoring, but also include training in NMD topics of disease mechanisms, pathophysiology and expected disease progression. Additional topics include:

✓ Good clinical practice (GCP).
✓ Good documentation practice (GDP).
✓ Knowledge of clinical research practice and study design.
✓ Internal validity risks and best management practices.
✓ Roles and responsibilities as a research/clinical evaluator and as key stakeholders in COA administration.
✓ Standardized training plan in core COAs used in each subset of NMD.

Advanced training to reach a proficient level should build upon the basic training concepts with increased applied learning skills to cement critical thinking skills required for assessing challenging or complex patients (Figure 2). Through applied situational learning, regular delivery of COAs, and exposure to patients with NMD, proficient, and expert learners should recognize similar characteristics/patterns across COA administration/scoring and NMD to increase fluid tacit knowledge.

Studies show that clinical experience is only one variable in accuracy and improved scoring of COAs. The most significant factor in improved accuracy in scoring COAs is CE foundational knowledge, familiarity with patients in NMD and frequency of training sessions. Targum (2006) found that with at least one training session, CEs of all clinical experience levels improved, but those who participated in 5 or more training sessions performed significantly better than those who attended only 1 session. Multiple training sessions should continuously build on the CEs current skill level, utilizing different modes of teaching. Training should consist of 2 parts: 1. Didactic learning 2. Applied learning (Table 2). For performance based COAs where quality of movement is scored, this education model increases familiarity with scoring the COAs and accuracy in performance. Cusick et al. (2005) found that training was associated with higher perceived level of performance and familiarity with the test items, improved reliability, decreased error, and improved internal consistency of test administration. Training reduced personal interpretation and allowed for understanding of applied theoretical constructs of each item which improved and standardized method of test item scoring.

**TABLE 2:** Examples of didactic and applied education.

**Didactic education**	**Applied education**
Conceptual knowledge: Scale development, Scale construct, COA overview, Review of manual of operations from item-by-item basis highlighting key points	Reliability: May be performed with video review or in person. Hands on lab to improve test administration and handling techniques ie: dolls, patients
Discussion of scoring construct	Practice in-person with volunteers with NMD, or using simulation
Review of video to demonstrate items or live demonstration	Video Review and discussion on scoring: Polling, Quizzes to facilitate discussion
Training in the study specific NMD	Quality control video review and feedback to enhance test administration throughout study.

Most studies focus on didactic learning, typically provided at only one investigators’ meeting. This provides understanding of the protocol, objective of the COA and a general overview of the known natural history of the disease. Cusick et al. (2005) found that this was insufficient for retention of knowledge and accuracy of administration of COAs. As didactic learning only provides conceptual knowledge on scale development, further in-depth instruction should cover essential components of the assessment and highlight common mistakes in administration and scoring.

Applied learning typically has a more hands-on approach that may be done in person through different methods (Table 2) that improve the CEs critical thinking skills and translation of information. This can ensure the CE is adhering to the standard administration from the COA manual, critically think through patient scenarios, and clarify vague or contradictory information as needed. Several studies have applied this type of learning through hands-on training, video review and reliability (Personius et al., 1994; Escolar et al., 2001; Ditunno et al., 2005; Mayhew et al., 2007; Steeves et al., 2007; Glanzman et al., 2018; Krosschell et al., 2018).

#### Qualification Requirements of Clinical Evaluator

Factors to consider regarding CE qualification during site selection include pre-determined minimal educational requirements, clinical experience and training required to perform and score COAs accurately. Since many COAs measuring treatment efficacy are function and performance-based assessments, PTs are a logical choice to serve as CEs. Since they receive extensive training in functional anatomy, physiology, and biomechanics as well as the psychometric properties of testing. They develop astute observational and palpation skills, which are necessary for assessments of strength and function. In addition, PTs have the skills to analyze movement and ensure that the quality of movement is aligned with the test objective. Similarly, PTs are skilled in motivating patients to perform movements and activities that may be challenging but required for a valid assessment, and can support with hands-on skills crucial to positioning for testing. Although most evaluations are currently performed by a licensed PT, these guidelines can also be used to instruct other healthcare professionals with similar backgrounds and sufficient documented experience. However, it is important to note that training and applied practice will require additional time and effort if a healthcare provider does not have the necessary foundational knowledge. Planning and providing for ample time for both didactic and applied practice opportunities is key to cementing knowledge and achieving valid and reliable COA administration and scoring.

A centralized process for training in multi-site clinical trials is essential for consistency in standardizing COA administration and data collection. With global diversity in CE experience and knowledge of COAs, centralization of the training process ensures consistency in who may perform COAs, current level of education, experience, acquisition, and maintenance of skills throughout the duration of the study (Kobak et al., 2004).

#### Training for Novice Clinical Evaluators

Training at the novice level should introduce the theory and development strategy behind disease-specific standardized COAs. Didactic aspects of the training could include video review in addition to the essential components of administering and scoring assessment (Table 3). This might be accomplished as part of an investigator meeting. The routine use of standardized COAs in the clinic provides objective monitoring of patients and improves clinical decision-making (Figure 1). Clinical data must be of the same high quality and rigor as in clinical trials as it may later be collated to support treatment efficacy comparing trial data to the natural disease progression. The novice level training will provide a strong foundation for the CE to acquire experience.

**TABLE 3:** Training plan overview.

**Foundational knowledge education plan**	
Trainer	COA Expert Physical Therapist Trainer
Trainee	Qualified Physical Therapist or comparable professional
Prerequisites	Preferred NMD specialty clinic experience COA experience for clinic or trials
Training Objective	Entry level knowledge on disease process, implementing and interpreting COAs and research best practice
Core Content	• Didactics of NMD disease pathophysiology • Biomechanics of movement • Applied: Hands-on lab and video review of COAs and functional scoring scale, Reliability with video

#### Establishing Initial Reliability

Evidence of an acceptable level of COA reliability should be achieved by the CE following didactic training, but prior to conducting any study assessments. Reliability should (1) Be established between the COA experts (considered the gold standard training evaluator), (2) Involve all study CEs responsible for COA administration, (3) Follow a reliability plan with pre-established objective minimal criteria. The criteria should be based on the standard error of measurement for each assessment if available. Ideally, reliability testing should consider inter-rater reliability which tests the CE’s ability to administer and score the test consistently and can be done through video evidence or by assessing a live patient (Glanzman et al., 2018). In-person reliability is essential but requires understanding of fatigue and motivational variables that may impact COA performance.

Studies have also successfully used video reliability as part of annual and refresher trainings in clinical trials in NMD (Glanzman et al., 2018; Krosschell et al., 2018). It is important, however, to ensure videos are of sufficient quality with appropriate camera angles to promote accurate scoring. For studies and clinical trials, intra-rater reliability may also be established utilizing the study design. These visits may be integrated into a screening and baseline visit to compare intra-rater reliability.

To demonstrate and document knowledge acquisition, CEs typically must pass a written or verbal quiz of the material. For administration competency, the CEs should be evaluated on a pre-determined level of scoring agreement with the COA expert. In addition, it is highly recommended that inter-rater reliability between CEs working in the same institution is assessed to ensure consistency. Programs should consider having opportunities and time built into budgets and schedules to ensure CEs have the resources needed to monitor and maintain reliability within their own site through periodic simultaneous evaluation of the same patient(s).

#### Testing Methods and Long-Term Monitoring of Clinical Outcome Assessment for Quality Assurance

Frequency of training throughout the study for knowledge acquisition and skill retention should be pre-determined as part of the training plan. After training, monitoring for quality control of COAs, calibration and CE drift is important (Kobak et al., 2004; Samuels et al., 2019). Different approaches have been used for re-training in clinical trials including annual and quarterly trainings where the focus is to highlight common errors seen in the quality control data or video review. Confirmation trials have also used video review and feedback throughout the trial to provide immediate advice through a “buddy” system that promotes ongoing learning and consistency throughout a trial. This approach facilitates open communication and a supportive relationship between the COA expert trainer and the CE to enhance the learning process.

Retraining should focus on knowledge retention and building, not a repeat of the original didactic information. Considering the fact that many NMDs are considered rare or ultra-rare, there should be a focus on learning over time as expertise will develop with repeated CE exposure to patients with these specific conditions. This may be done through traditional video review and discussion of participants from the study, particularly if there is a change in phenotype. Other studies have utilized technology for enriched trainings utilizing interactive web portals to enhance knowledge acquisition (Samuels et al., 2019). A multi-site study for a depression trial that utilized traditional and web-based tutorials showed improvements in didactic and applied skills that enhanced accessibility, training and cost effectiveness (Kobak et al., 2006). Consideration regarding CE burden and different modes of training should be taken into account, including hybrid models of web-based, videoconference and in-person trainings. The ongoing, periodic retraining is relevant for longer-term studies to ensure accuracy in COA administration and reliability.

#### Considerations for International Multi-Site Studies

Factors such as linguistics, behavior and cultural differences may impact COA administration, knowledge acquisition, and interpretation for global trials. Consistency with training is key to ensure accuracy and standardization. We recommend that the same manuals, study worksheets and training materials be translated into the site’s native language and presented to all sites within the study. The material must be translated and back translated in consultation with a COA expert to make necessary adjustments based on clinical interpretational linguistic differences. To ensure accuracy of translation and understanding of COAs, we also recommend real time, or simultaneous translation for both the didactic and applied education series. From our experience, errors in translation have been clarified during in-person trainings that included simultaneous translation for international studies. It is important to consider educational requirements and scope of professional practice as they can vary significantly among international medical and allied health professions. It is important to take this into consideration when determining the training requirements and quality assurance for COAs.

## Discussion

With the current landscape of NMD it is critical to standardize study training to ensure robust data in clinical trials and accurate long-term monitoring in clinic, post marketing, and natural history studies. Currently in studies involving NMD, COA training varies across different disease cohorts but it usually consists of a combination of didactic teaching of COAs and disease presentations, video review of COA administration followed by discussion, demonstration and practice of performing the COAs. These usually occur at investigator meetings or on-site training visits. Intra-rater reliability may be established in some protocols as part of the study design and inter-rater reliability may be established via video review or in person patient testing (Brooke et al., 1981; Florence et al., 1984; Personius et al., 1994; Escolar et al., 2001; Mayhew et al., 2007; Krosschell et al., 2011, 2018; Glanzman et al., 2018).

This guideline utilizes the extensive experience from global expert physical therapist/clinical evaluator trainers to provide recommendations for training and education in COA administration. Quality COAs impact the interpretability of data that is used as efficacy endpoints in NMD trials and guides clinical decision-making and planning treatment options. As part of a Clinical Globalization of DMD Outcomes project, we developed a DMD training guideline and standard operating procedures (Supplementary Material).

The accuracy of COA administration relies on many variables including existing knowledge, experience and type of training completed. Targum (2006) found that clinical experience was not statistically impactful in the accuracy of COA scoring in neurology trials but identified that the number of training sessions was the more significant factor. All CEs need comprehensive and ongoing training regardless of clinical experience. Frequent training throughout a study improves familiarity with COAs but is particularly important in trials that have significant impact in changing the pre-treated phenotype. CEs must acquire skills to accurately and reliably administer COAs with different types of patients within the construct of test.

Clinical care and monitoring have improved with clinical trial experience and training particularly at sites who lacked standardized methods for COA administration. Early harmonization of manuals and standard operating procedures in outcomes from DMD trials have provided unique opportunities to combine and analyze data from various sources. The Collaborative Trajectory Analysis Project (CTAP) leverages natural history, placebo and real-world clinical data to understand the heterogeneity in disease progression and identify prognostic factors to loss of key clinical milestones (Mercuri et al., 2016; Goemans et al., 2020). Efforts, such as these in COA administration, are essential for rare and ultra-rare NMD as they allow for data modeling that may impact clinical trial design, characterize disease trajectories, assess meaningful change, and be used for external controls and interpretation of outcomes.

There is increased cost associated with comprehensive, standardized and effective training. This may be a reason for the lack of adoption of universal education and training guidelines of COA. However there is a much higher financial and emotional cost of a failed trial. Comprehensive training reduces variability in COAs which may impact study design in estimation of expected effect size and power. Several studies have found that the training method of COAs may have potential impact on failed trials in behavioral sciences and Central Nervous System Disorders (Mulsant et al., 2002; Kobak et al., 2004; Jeglic et al., 2007). With enormous costs of large multi-site international trials in rare disease, it is imperative to appropriately document methodological approaches to improving accuracy and reliability of COAs in clinical trials and in post marketing studies. Cusick et al. (2005) assessed “trained” and “untrained” CEs for the evaluation of upper limb function and found that even if provided with a manual for the study, none of the CEs read the manual, and only the trained group reviewed the details of the manual during the training. In a fluoxetine study, lack of inter-rater reliability and training contributed to large variability in depression scale scores and such was hypothesized as a major factor in the failed trial (Mulsant et al., 2002).

Since the initial MDA multi-site DMD training study (Brooke et al., 1981), aspects of the training model described in this guideline have been administered across different NMD studies from natural history to large multi-site international gene therapy trials with good success. We have built on these previous experiences to refine variables that result in improved accuracy, quality and delivery of COA training.

• Training plan must be pre-determined to include qualifications of CE, COA training content materials, and training approach and methods including re-training for longitudinal studies.
• Manuals for the validated COA should be harmonized across the study and consistent with published material.
• Previous CE experience and training with COAs should be documented to decrease redundancy in training across disease groups and adapt education material to meet individual learning styles and needs.
• Timely and regular feedback is essential to optimize learning and improvement of COA quality.
• Engagement by using technology allows for knowledge transfer, uptake and retention.
• Intra-rater reliability is often higher than inter-rater suggesting the same CE per patient throughout the trial especially at key study milestones is important to consider.
• Pre-Study site selection should include CE training experience so this may be integrated into a study training plan to provide sufficient timelines for CE training, preventing delays in study start up and recruitment.

One of the limitations of this guideline is that we do not have empirical data to support recommendations as results of training and reliability across programs is often proprietary and not reported. This consensus is based on the collective experience of global COA expert trainers in the NMD field. However, one of the strengths of this guideline is the wealth of experience of the authors from design of original natural history studies for Duchenne Muscular Dystrophy over 40 years ago to the current gene therapy trials where we gained vast amounts of experience in a very different pre vs. post treatment phenotype. These learnings are important as the NMD trial landscape shifts from using disease specific COAs designed based on pre-treatment compensations and now moving to a treatment era that will include combination therapies resulting in changes in function and disease progression. We acknowledge that the authors are trainers and advisors on many large NMD clinical trials and may be viewed as possible conflicts of interest. However, our experiences and recommendations align with published studies in behavioral and neurological research on the importance and need for centralized, standardized training to ensure reliable administration and scoring of COAs (Kobak et al., 2004; Targum, 2006; Jeglic et al., 2007; Walker et al., 2014). We have collated our experiences to provide a practical approach toward this goal and the contents of this guide may be used in context of the specific NMD, purpose and phase of the trial. Many trials have primary and secondary efficacy endpoints based on COAs performed by CEs therefore training methods must be thoughtfully and rigorously employed to ensure longstanding high data quality across diverse clinical and research settings. We hope that the collective lessons learned summarized in this consensus-based guideline will result in quality and consistency in COA administration allowing for improved confidence in data interpretation to further advance the clinical understanding of the changing landscape in neuromuscular disorders.

### Advanced Learner

Individual with experience and comfort administering and scoring common COA; selects appropriate COA for diagnosis; assesses challenging and complex patients and cohorts.

### Clinical Evaluator

Individual, often a physical therapist, who will perform clinical research assessment of outcomes that will be implemented in clinical trials or in the clinic. This individual will have training in implementation of clinical research assessment of outcomes in a standardized manner following good clinical practice.

### Clinical Outcome Assessment

Based on the Food and Drug Administration (FDA) Clinical Outcome Assessments measure a patient’s symptoms, overall mental state, or the effects of a disease or condition on how the patient functions. COAs may be used to determine whether or not a drug has been demonstrated to provide treatment benefit. Treatment benefit may also be defined in terms of a safety benefit compared to other treatments. A conclusion of treatment benefit is described in labeling in terms of the concept of interest, the *thing* measured by the COA^1^.

### Expert Learner

Individual who understands COA construct, critically analyzes psychometric properties; develops and validates COA when needed.

### Inter-Rater Reliability

The concordance of agreement between different CEs and/or MP. It is used to assess the degree to which different evaluators agree in their assessment decisions.

### Intra-Rater Reliability

The concordance of agreement of a given CE and/or MP. It is the degree of agreement among repeated administrations of a clinical evaluation performed by a single evaluator.

### Clinical Outcome Assessment Expert Trainer

Individual who has expertise in COA development and training. Exhibits expert-level skill and knowledge; Conveys and teaches COA to learner(s); adapts teaching style; identifies and conducts remediation/retraining.

### Novice Learner

Individual with foundation of GCP, GDP, clinical research practice, internal validity risks, best management practices, roles and responsibilities, basic outcome administration, and scoring training.

### Neuromuscular Disorders

Disorders that impair functioning of muscle (directly or indirectly) and originate from: anterior horn cells, nerves, neuromuscular junction, muscle, and peripheral nervous system pathology.

### Post Marketing Surveillance

Post marketing surveillance refers to the collection of subject data after the approval and marketing of a pharmacological therapy. Continued collection of safety data that may include unexpected side effects and continued efficacy of treatment takes place during this period. This data is usually collected at clinical sites treating patients with the newly licensed or conditionally licensed therapy. Accelerated approval mechanisms are now shifting confirmatory clinical research studies (phase 3) into the post-marketing arena. Post marketing study conditions are set by the regulatory authority granting the license to market the therapy.

### Qualification Criteria

Minimum educational, professional and experiential considerations required for COA administration or training.

### Clinical Evaluator Drift

Refers to the decreased consistency in test administration over time.

### Training

An educational process to determine precise, accurate and reliable administration and scoring of COAs.

## Author Contributions

TD, KJK, MKJ, LN, LNA, KE, EM, KR, LPL, AM, JF, WK, CRS, and ME contributed to the conception or design of the work or the acquisition, analysis or interpretation of data for the work, drafted the work or revised it critically for important intellectual content, provided the approval for publication of the content, and agreed to be accountable for all aspects of the work in ensuring that questions related to the accuracy or integrity of any part of the work are appropriately investigated and resolved. All authors contributed to the article and approved the submitted version.

## Conflict of Interest

TD has served on medical advisory boards and/or consultant for Scholar Rock, Genentech, F. Hoffman La Roche, Biogen, Sarepta, Novartis, Solid Biosciences, Dynacure, Dyne, and Audentes Consultancy also through ATOM International and Trinds (Biomarin, Pfizer, Solid Biosciences, Sarepta, and Astellas). She has received research grant support from Ionis. KJK receives consulting fees from Cure SMA, ASPA Therapeutics, and Biogen and Honoraria from Stanford University and Cure SMA; Advisory Board member of Biogen and Cure SMA; Roche; Grant funding: Academy of Pediatric PT, APTA; subcontracts from Lurie Children’s for Biogen, Cytokinetics, Avexis, and Scholar Rock and NIH iAcquire clinical trial. MKJ provides consultancy services for the following companies: ATOM International (covers consultancy services provided to Amicus Therapeutics Pty., Ltd., Ascendis Pharma, Biomarin, Catabasis, Faraday, FibroGen, Genethon, Italfarmaco, NS Pharma, Pfizer, PTC Therapeutics, QED Therapeutics Ltd., Reveragen, and Sarepta Therapeutics). MKJ has received payment for participation on Advisory Boards for F. Hoffman La Roche AG, PTC Therapeutics and fee support for Ph.D. studies from the Jain Foundation. LN has served on Medical Advisory Boards and as a consultant for Sarepta, Pfizer, Biogen, Novartis, Scholar Rock, Genentech, and F. Hoffmann-La Roche. She served as a member of ATOM from 2015 to 2020. LNA provides consultancy services through ATOM International for the following companies: Amicus Therapeutics Pty., Ltd., Catabasis, Genethon, Italfarmaco, NS Pharma, Pfizer, and PTC Therapeutics; reports royalties and other support from Sarepta Therapeutics; royalties for licensed technologies; other support from Novartis Gene Therapies; and advisory board for Biogen. KE has received personal compensation for serving on advisory boards and/or as a consultant for Ionis, Biogen, Acceleron, Fulcrum, Avidity, PTC, F. Hoffman-La Roche, and the Myotonic Dystrophy Foundation. KE has received personal compensation for serving as a speaker from Cure SMA, FSH Society, and Ology. She has received research/grant support from the CMTA. EM has served on medical advisory boards and/or consultant for Scholar Rock, F. Hoffman La Roche, Italfarmaco, Biogen, Sarepta, Novartis, Avexis, and PTC Therapeutics. KR provides consultancy services (training in clinical outcome measures and quality assurance implementation for clinical outcome assessments) for the following companies: ATOM International (under ATOM consultancy services provided to Amicus Therapeutics Pty., Ltd., Ascendis Pharma, BioMarin, Catabasis, FibroGen, Italfarmaco, NS Pharma, Pfizer, PTC Therapeutics, QED Therapeutics, Ltd., Sarepta Therapeutics, and Summit Pharmaceuticals International). KR also provides independent consultancy services to Biogen and F. Hoffman La Roche AG and receives payment for participation on Advisory Boards and publication steering committees and for assisting to deliver education initiatives for Biogen and F. Hoffman La Roche AG. LPL provides consultancy services through ATOM International for the following companies: Amicus Therapeutics Pty., Ltd., Catabasis, Genethon, Italfarmaco, NS Pharma, Pfizer, and PTC Therapeutics; reports royalties and other support from Sarepta Therapeutics; royalties for licensed technologies; other support from Novartis Gene Therapies; and advisory board for Biogen. AM provides consultancy services for the following companies: ATOM International (covers consultancy services provided to Amicus Therapeutics Pty., Ltd., Ascendis Pharma, Biomarin, Catabasis, Faraday, FibroGen, Genethon, Italfarmaco, NS Pharma, Pfizer, PTC Therapeutics, QED Therapeutics Ltd., Reveragen, and Sarepta Therapeutics). AM has received payment for participation on Advisory Boards for F. Hoffman La Roche AG, PTC Therapeutics. AM also provides independent consultancy services to Biogen and F. Hoffman La Roche AG. ME is Managing Director of ATOM International Limited and provides consultancy services for the following companies: Amicus Therapeutics Pty., Ltd., Ascendis Pharma, Biomarin, Catabasis, Capricor, Denali Therapeutics, Faraday, FibroGen Inc., Genethon, Italfarmaco, Lysogene, Modis, NS Pharma, Pfizer, PTC Therapeutics, QED Therapeutics, Ltd., Reveragen, Sarepta Therapeutics, and Solid Biosciences. The remaining authors declare that the research was conducted in the absence of any commercial or financial relationships that could be construed as a potential conflict of interest.

## Publisher’s Note

All claims expressed in this article are solely those of the authors and do not necessarily represent those of their affiliated organizations, or those of the publisher, the editors and the reviewers. Any product that may be evaluated in this article, or claim that may be made by its manufacturer, is not guaranteed or endorsed by the publisher.

## Footnotes

1
https://www.fda.gov/drugs/development-approval-process-drugs/drug-development-tool-ddt-qualification-programs

## Abbreviations

CE: clinical evaluator
ClinRo: clinician-reported outcome measure
COA: clinical outcome assessment
CRF: case report form
DMD: Duchenne muscular dystrophy
EK: Egen classification
FDA: food and drug administration
EMA: European Medicines Agency
GCP: good clinical practice
GDP: good documentation practice
HHM: hand held myometry
IM: investigators meeting
MDC: minimal detectable change
MMT: manual muscle testing
MP: master physiotherapist
NMD: neuromuscular disorder
NSAA: north star ambulatory assessment
ObsRO: observer-reported outcome measure
PerfRO: performance outcome measure
PRO: patient reported outcome measure
QC: quality control
QMT: quantitative muscle testing
ROM: range of motion
SOP: standard operating procedure
6MWT: 6-min walk test
PFT: pulmonary function testing.
