# Supplementary material for: Consensus Guidelines for Improving Quality of Assessment and Training for Neuromuscular Diseases
Source: Front Genet. 2021 Nov 10;12:735936. doi: 10.3389/fgene.2021.735936 (PMC8631528; doi:10.3389/fgene.2021.735936)
Supplement: Supplementary file 1 [file Data_Sheet_1.pdf]

Supplementary Material

Consensus guidelines for DMD Outcomes Training

This consensus document was developed during a 2015 meeting among 14 healthcare professionals with expertise in clinical trial design and clinical outcome assessments (COAs) in Duchenne Muscular Dystrophy (DMDs). This document is intended to act as a guide for those wishing to utilize clinical outcome assessments (COAs) for clinical trials in DMD. They are intended to be viewed as a guide only. These guidelines are not intended to endorse any commercial product or entity. While we hope they prove useful, we do not endorse these as the industry standard.

Table of Contents

1 SELECTION OF STUDY SITE CES ... 2

1.1 SITE CE QUALIFICATION RECOMMENDATIONS ... 2
1.2 CE SELECTION AND TRAINING OF NON-LICENSED PHYSICAL THERAPISTS ... 3

2 BASIC-FOUNDATIONAL CE TRAINING PROCESS ... 3

3 ADVANCED TRAINING PROCESS ... 4

4 SUGGESTED TRAINING PATHWAYS FOR NOVICE AND EXPERIENCED CES ... 5

4.1 PATHWAY 1: TRAINING FOR NEW CES VIA COA SPECIFIC TRAINING SESSIONS ONSITE ... 5
4.2 PATHWAY 2: CE TRAINING VIA STUDY-SPECIFIC INVESTIGATOR MEETING (IM) ... 6
4.3 PATHWAY 3: CE TRAINING VIA WEBINAR FOR EXPERIENCED CES. ... 6
4.4 STANDARD TRAINING/RELIABILITY REQUIREMENTS ACROSS ALL PATHWAYS: ... 7

5 RETRAINING IN COAS ... 8

6 FREQUENCY OF CE RECERTIFICATION ... 9

7 QUALITY ASSURANCE OF COAS IN CLINICAL TRIALS ... 10

... 14

8 THE ROLE OF THE EXPERT COA TRAINER (ET) ... 15

9 EXPERT TRAINER CONSULTANCY TO THE STUDY SPONSOR ... 15

a. Study Design/Outcome Measure Selection ... 15
b. Clinical Evaluator (CE) Training and Study Support ... 15
c. Case Report Form (CRF) Development ... 16
d. Development of Electronic Database Capture (EDC) for COAs including advising on data checks ... 16
e. Participation in Investigators Meetings (IMs) ... 16
f. Data Analysis ... 17

10 EXPERT TRAINER AS AN ONGOING SUPPORT TO SITE CES ... 17

11 EXPERT TRAINER ROLE IN ONSITE TRAINING ... 18

12 EXAMPLE OF COA IN DMD ... 19

1 Selection of Study Site CEs

This section of the guideline provides recommendations for the selection of site personnel who will act as CEs, conducting COAs for clinical trials of DMD. Study sponsors should recruit licensed/registered physical therapists (PTs) to become certified site CEs. PTs are valuable in clinical trials of neuromuscular diseases because of their expertise in movement kinematics, muscle activation/coordination, and biomechanics which are essential to accurately evaluate mobility, especially in individuals with impairments in the neuromuscular system.

During a trial, a primary CE will be responsible for COA testing, while a second CE should be assigned as back-up in the event a back-up CE is called to act as the primary CE. These personnel should be identified during site feasibility visits. The use of a questionnaire to obtain information regarding the candidate CEs professional qualifications and experience in COAs relevant to the study is encouraged. The questionnaire should be reviewed by a clinician appropriately qualified to determine whether the respondent has the appropriate skill set and qualifications to conduct evaluations for the study and make judgement as to their experience level. The site CEs will perform and document COAs for study subjects following Good Clinical Practice (GCP), as outlined in the clinical trial protocol designed by the study sponsor.

1.1 Site CE Qualification Recommendations

- Minimum of undergraduate degree in physical therapy or equivalent allied health professional
- Licensed/registered physical therapist or equivalent
- Required skillset for COA administration

Completion of comprehensive COA training and demonstration of intra-rater and inter-rater reliability.

1.2 CE Selection and Training of Non-Licensed Physical Therapists

The professional qualifications of CEs may vary depending on the study, sponsor, geographical region or site. It is highly recommended a licensed/registered physical therapist (PT) be certified as the site CE. However, some sites may choose to use a non-licensed physical therapist as their site CE, or other allied health or medical personnel. Under these circumstances, it is suggested these individuals have a comprehensive background in neurology and/or rehabilitation and/or a degree and extensive experience in DMD assessments. This is to ensure all CEs have a foundation of knowledge and expertise to ensure consistent quality assessments.

2 Basic-Foundational CE Training Process

This section of the guideline is designed to ensure efficiency in CE preparedness for COA testing for clinic purposes, rese arch and post-registration testing.

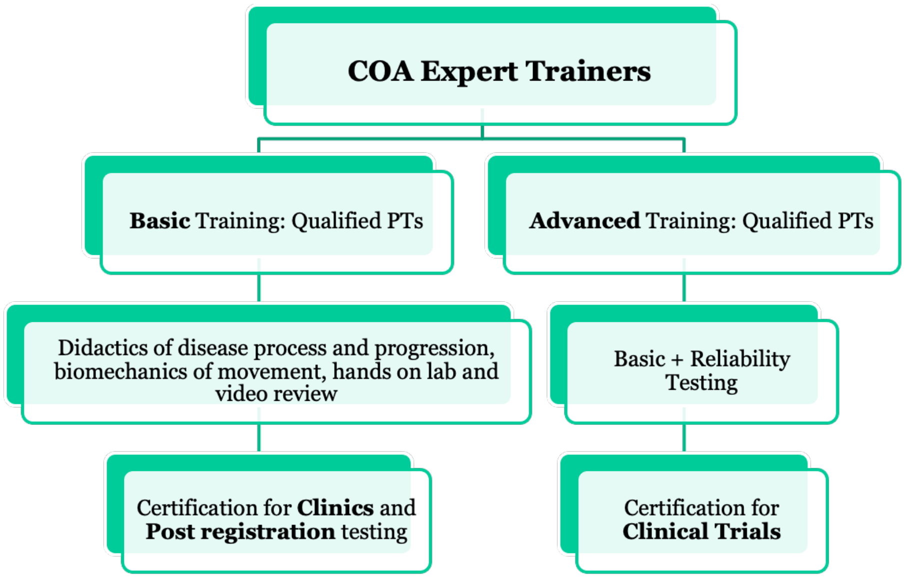

Figure a: Certification Process for COA training

Basic foundational training consists of didactic training on the history and scale development, administration and scoring guidelines for the COAs. CEs will be provided training manuals and worksheets for the COAs. Training may be done in person or via web-ex for the COAs. After completion of didactics training, the CEs will be required to pass an online quiz to ensure understanding of the material.

Following basic-level CE certification training via one of the above pathways, the CE will:Receive a certificate of documenting participation in training

- The CE will sign a document stating understanding and review of the COA manuals and worksheets, as well as acknowledge they will perform all assessments in accordance with the CE manual.

3 Advanced Training Process

Advanced training is designed to ensure efficiency in CE preparedness for COA testing in clinical trials of DMD by eliminating redundancy in training requirements for CEs who have had Basic training. The objective is to build on the skills to improve critical thinking and decision making in a consistent manner.

To allow for a range of prior experience and involvement in clinical trials of DMD, expedited start up and quality training will be based on CEs obtaining basic foundational knowledge that may be documented through previous trainings of the COA from involvement in other DMD clinical trials. Outlined below are the three paths for new or experienced CEs to become certified and clinical trial ready. All three pathways outlined below will lead to CE Advanced Certification.

4 Suggested training pathways for novice and experienced CEs

It is important to ensure new CEs are sufficiently trained to conduct COAs in a reliable and valid manner. It is also important to maximize efficiency and applied knowledge in COA training for advanced CEs by eliminating redundancy in their training requirements and build critical thinking applications. To allow for a range of prior experience and involvement in clinical trials of DMD, expedited CE training should be an option for experienced CEs. Outlined below are the 3 training pathways for new or experienced CEs to become certified and clinical trial ready. Regardless of prior qualification, all CEs must undergo study specific COA training via one of the 3 routes described below. We strongly recommend experts in COAs to conduct training of CE's. Cascade training by those previously trained in COAs should not occur under any circumstances.

4.1 Pathway 1: Training for new CEs via COA specific training sessions onsite.

This pathway is designed for CEs with no prior COA training and/or no experience in DMD. New CEs will receive in-person training conducted by a PT expert trainer (ET) at the study site. This training should consist of didactic instruction and hands-on practice, preferably with a patient with the condition being studied in the trial. They should also participate in inter-rater and intra-rater reliability testing. More specifically, we suggest new CEs are provided with didactic training on the DMD condition being studied in context with the DMD natural history progression, an introductory presentation covering aspects of the protocol relevant to CEs and the importance of standardization in the administration of COA. Presentations of each COA included in the study should include the history of its development and a detailed review of administration and scoring guidelines. To check information retention following didactic

training, we suggest requirement to pass didactic knowledge quiz to document understanding of scale development and administration in the protocol specific disease phenotype..

4.2 Pathway 2: CE training via study-specific investigator meeting (IM)

This pathway is designed for CEs who have minimal to no prior experience evaluating DMD patients using the COAs included in the study. Training should be customized to experience levels of the CE attendees. CEs with no prior training on COAs would require education as part of the basic training including in depth didactics to review the manual and item by item instruction. This would require novice CEs to attend an additional session to ensure that all CEs attending an IM training have similar background education. IMs provide an excellent opportunity to provide training to a large group of CEs. The presence of multiple trainers allows for the possibility of breakout groups organized on the basis of CE experience with DMD and with COA administration.

At the IM the CE should be provided with didactic and practical training. This pathway also allows CEs to establish inter/intra-rater reliability of site CEs and therefore qualifies the CE to perform trial-specific evaluations. This training method ensures administration of DMD COAs in a consistent manner at IMs utilizing CE experience to customize training needs for quality COA administration.

4.3 Pathway 3: CE training via webinar for experienced CEs.

This pathway is designed for CEs with extensive COA experience but unable to attend the study IM. Experienced CEs have likely undergone previous training to perform trial-specific evaluations. They may be qualified to perform study-specific training if:

i. Inter-rater reliability training has been documented within the last two years

ii. The CE has been performing **at least** two relevant COAs per quarter documented as part of the study or in clinic.

Study specific training would typically provide a protocol review appropriate to CEs, review the COAs and the order they will be assessed at study visits and discuss any differences that may exist between this study and others the CE may have been trained in. Should the CE feel they would like to have their knowledge refreshed or clarify their understanding of the COAs this should be done.

Experienced CEs have typically demonstrated evidence of reliability in the administration of the COAs, however if more than 2 years has lapsed since their last documented reliability testing, a trainer visit will be required for the site CEs to update reliability requirements.

4.4 Standard training/reliability requirements across all pathways:

Regardless of the training pathway being followed CEs should be provided supplementary training materials including manuals and worksheets for the COAs. These documents should be translated into the local language of the CE if their first language is not English. A translator should be engaged to provide simultaneous translation during training and reliability in the event the trainer does not speak the local language of the CE.

Regardless of experience all CEs should meet the interrater and intra-rater reliability thresholds throughout the clinical trial as established by the *minimal reliability standards* for a given outcome. The *minimal reliability standards* should be based on previously published studies on outcome reliability and Minimal Detectable Change (MDC).

On completion of their training, CEs should sign a document confirming they have reviewed and understood the COA manuals and worksheets and acknowledge they will perform all assessments in accordance with the CE manual. Certification acknowledging the CE has

completed all elements of training should be issued as proof of training for regulatory purposes. New CEs will achieve novice level certification. While experienced CEs will achieve certification at an advanced level.

5 Retraining in COAs

It is recommended that CEs are retrained on a regular basis as gradual deviations or drift from the original study COA standards are frequently observed over time and have the potential to impact the quality of study data. CEs should be required to complete COA refresher modules for retraining to be granted. The retraining process will depend on the type of training pathway the CE started on (Figure b). Retraining should be based on site CE testing quality and standardization based on either video or data review, frequency of assessments and standard need for general re-training to decrease drift of knowledge and COA administration associated with time, particularly for those who do not routinely perform COAs. Refresher training is recommended if study enrollment is delayed, and no subject evaluations are completed within 3 months of initial training. This should be established in the COA Training plan

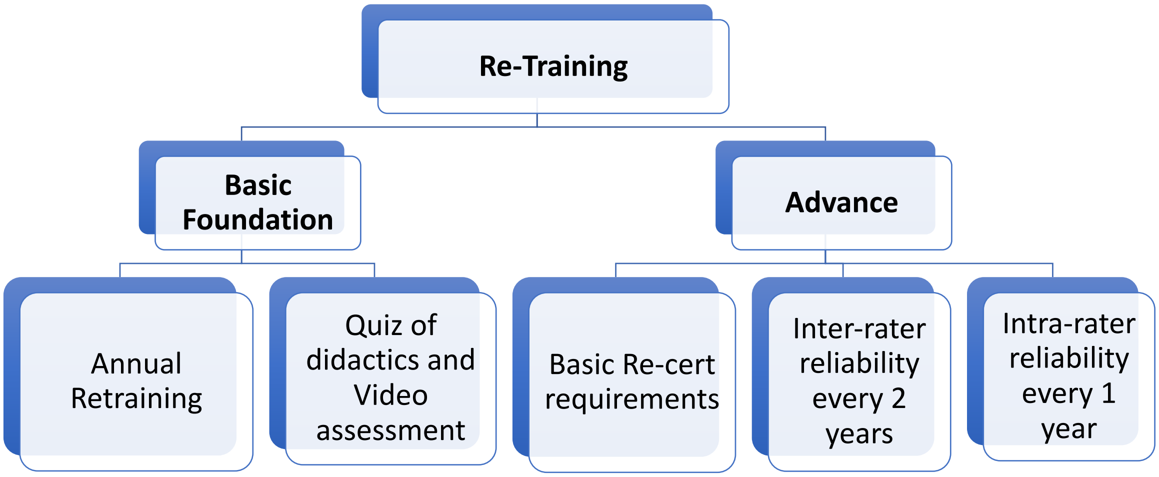

Figure b: CE Re-Training Process

6 Frequency of CE recertification

Studies longer than one year should require study-specific retraining via webinar at the very least one year after the IM or one year after the first subject's first visit in cases where recruitment is delayed. An allowance of 3 months between training and the first subject assessment should be permitted.

For CEs with novice level certification, one or both of the following knowledge-based training mechanisms may be implemented to maintain consistent testing quality.

- CE Quiz:
  - This is designed to test the knowledge of the CE regarding information outlined in the COA manual. If there are any discrepancies in the answers provided by the CE, then the specific member of the training team working with the site will follow up and clarify these misunderstandings to establish increased understanding of correct test implementation and scoring.

    Note: CE Quiz length and content may vary based on COAs. Quiz may include abbreviated review of video assessments or COA content
- Webinar:
  - This is designed to review COA standards of testing and review of frequently seen deviations in testing.

For CEs with advanced level training, they should:

- Satisfy the requirements for novice retraining outlined above.

- Have inter-rater reliability testing performed every two years. CEs must meet the minimum inter-rater reliability requirements per study protocol.
- Perform intra-rater reliability testing at least once per year and at the beginning of every new study according to study protocol. This may be performed as part of study specific testing protocol during patient screening and baseline visits.

Alternative routes for CE retraining may be documented through satisfactory video reviews or participation in appropriate investigator meetings or relevant inter-rater reliability testing performed as part of on-going clinical trials. Different routes of re-training will be based on study specific protocols.

7 Quality Assurance of COAs in Clinical Trials

COAs are frequently included as primary endpoints in clinical trials of DMD. We therefore encourage the sponsors of these trials to engage experts to advise in the selection of COAs and develop a quality control (QC) framework appropriate to their study design and duration. A robust QC framework around COAs will assist the sponsor in primary endpoint data is tightly controlled at the regulatory level.

We recommend an advanced quality control process be implemented for clinical studies where the primary COA endpoint is performed by CEs. This process will ensure quality of standardized COA testing for that trial. It is important to ensure candidate CEs are adequately qualified to conduct COAs at the standard required per the recommendations outlined in the early stages of this document. Evidence of education and training should be provided by the candidate CE. Additional information relating to clinic and clinical trial experience can be obtained via a questionnaire. It is imperative candidate study CEs are identified during site feasibility visits so their training needs can be rapidly determined and there be no delay to site initiation. Once they

are identified, CEs should receive training in the standardized administration of COAs by individuals considered expert in the COAs and disease. Training should always be tailored to the experience level of the CE. In this document, we have suggested three different training pathways and the type of training which should be included for each. This could be modified depending on the study.

In order to document CE Training, it is imperative a database is used to track the method and dates of CE training. Prior to study start up, a list of sites with their estimated dates of initiation can be helpful for trainers to prioritize and plan their training. This will reduce training redundancy, increase efficiency and decrease study start up time. It will also ensure that events such as reliability expiration can be flagged well in advance of when they occur. Regular meetings with the central study team are suggested to receive study updates, clarify training priorities and maintain an open line of communication.

All study CEs, regardless of experience or whether they are the primary or backup CE onsite, must be reassessed to ensure they continue to conduct COAs to the required standard. We recommend that CEs perform two or more COAs per quarter, according to study-specific requirements. This recommendation is to ensure a CE has enough experience assessing the relevant clinical endpoints for a trial. In addition, performing two or more COAs per quarter will provide the necessary practice for a CE in the event an extended period passes between training and actual test implementation.

Video review of COAs for quality control can prove very useful for ensuring assessments are being conducted in a standardized manner. If the sponsor wishes to include video QC in their protocol it is important to include video/photography consents and video review is mentioned in the study protocol. Further, a secure portal meeting data and privacy regulations should be used

to house the videos. The video review should be conducted by an expert ET. They will systematically review the video to ensure appropriate testing environment, test administration and that only certified CEs conduct study assessments. For example, video QC of a muscle strength assessments will take into consideration the following:

- ✓ Appropriate implementation of standardized testing positions
- ✓ Appropriate verbal testing instruction
- ✓ Level of motivation provided to patient
- ✓ Appropriate understanding and ability to manage compensatory mechanisms
- ✓ Appropriate documentation of compensatory mechanisms related to test scoring or validity

Feedback should be communicated to the CE in a timely manner (prior to the next test administration), via a video QC report. This should be translated in the instance the reviewers' first language is not the same as the CEs.

There may be instances where significant errors are detected upon video QC. An *unsatisfactory assessment* is defined as an invalid outcome measure resulting from a major deviation in the administration procedures outlined in the CE manual. These deviations are usually detected by the ET during QC video reviews. Two or more (≥2) unsatisfactory assessment reports will require in-person re-training with an ET. Examples of major deviations include:

- ✓ Performing North Star Ambulatory Assessment (NSAA) wearing shoes or orthosis
- ✓ Allowing the patient to sit during 6-minute walk test (6MWT)
- ✓ Allowing the patient to persistently touch the wall for support during 6MWT

In the event a major deviation is noted, the CE should be able to recognize and document the test is invalid or patient positioning is inappropriate for muscle strength/functional assessment testing. In the case an assessment is deemed unsatisfactory, leading to an invalid primary outcome measure, the following actions must be taken:

- ✓ An impartial ET Is to objectively review the video containing the potential deviation. The impartial ET should document their video review using the QC report template and all communication regarding the assessment should be archived.
- ✓ The ET will contact the study sponsor via email once QC review and report of findings are completed. Decision of impact and action plans will be reviewed and determined in collaboration with the sponsor.
- ✓ The reviewer will notify the study site of testing deviations through the QC report and directly contact the study sponsor via email and/or telephone.
- ✓ The ET will provide constructive feedback to the CE under review.
- ✓ If required, remediation via retraining is conducted. This could be done via telephone, web-based, or in-person. This must be performed prior to the CE's next visit.

There are often instances where *minor errors* that do not impact the scoring or validity of an assessment may be detected via video QC review. These errors should be documented in the comments section of the QC report and shared with the CE. Examples of minor errors include

- ✓ Forgetting to cross the patient’s arms during sit to stand in the NSAA
- ✓ Strength testing out of protocol order

✓ Incorrect order within a functional scale e.g., sit to stand after stand on one leg

Although minor errors might not impact overall assessment, it is important to improve consistency of test administration by a CE. Recurrent errors over several assessments or multiple errors within one assessment will lead to poor quality results. If that is the case, the QC reviewer will email the CE and study sponsor. CE re-training with ET may be arranged.

The use of video review at a subject screening visit (prior to baseline visit) is highly recommended. This will allow for assessment errors to be identified that would potentially affect the baseline data. For video QC and any associated remediation to occur between screening and baseline visits, the video should be uploaded, and review occur as soon after the assessment as possible. It is important the ET and study sponsors discuss the timeline requirements for video upload and review.

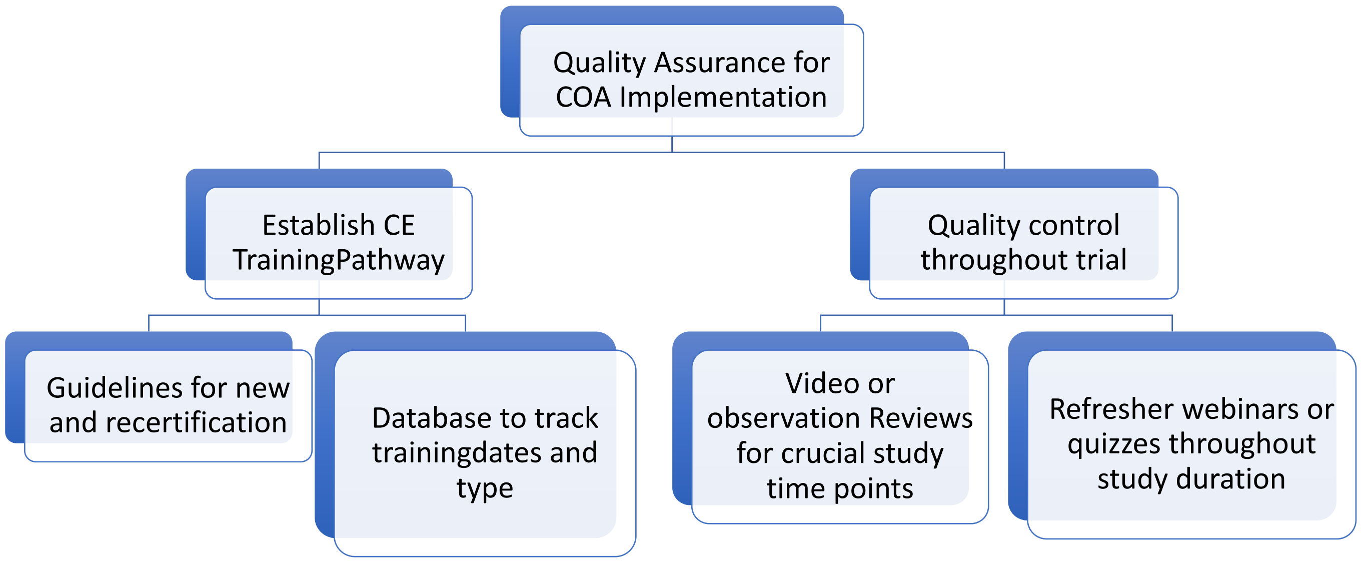

Figure c: General Overview of Quality Assurance

8 The role of the expert COA trainer (ET)

Expert Trainers have significant expertise in the development, administration and teaching of COAs and support the implementation and management of clinical trial readiness of CEs at a study site. The level of ET involvement may vary from study to study and should be outlined and agreed upon by the study sponsor and ET in the study design phase. This information and delegation of work should be outlined in the COA training plan prior to study start up. Outlined below are potential roles ET s may play to ensure quality in study implementation. Figure c illustrates ET responsibilities at different trial stages.

9 Expert Trainer consultancy to the Study Sponsor

a. Study Design/Outcome Measure Selection

   The ET may assist the study sponsor in the selection of outcome measures that are appropriate to quantify drug safety and/or efficacy. This selection is based on:

   - ✓ The investigational study aim(s)
   - ✓ The known or anticipated mechanism of action of the product under investigation

b. Clinical Evaluator (CE) Training and Study Support

   The ET should assist the study sponsor in developing the CE manual, study worksheets and training plan for the study. The CE COA training plan outlines important aspects of the trial, including:

   - ✓ CE roles and responsibilities
   - ✓ Reliability procedures and expectations
   - ✓ The process of quality oversight of COAs by the study ET

- ✓ ET /CE support system
- ✓ Standardized administration and scoring of all study assessments

c. Case Report Form (CRF) Development

The ET should assist the study sponsor in drafting and developing CRFs, which are designed to meet Good Documentation Practice (GDP) guidelines. These include:

✓ All relevant scoring	• Individual item scores<br>• Time to perform tasks<br>• Quality of movement rates
✓ De-identified subject information	• Study name<br>• Study identification number<br>• Visit number<br>• Visit date
✓ CE identification	• Name and signature

ETs should work in collaboration with the study sponsor to ensure the data forms related to COAs are comprehensive and consistent with electronic data collection.

d. Development of Electronic Database Capture (EDC) for COAs including advising on data checks

e. Participation in Investigators Meetings (IMs)

The purpose of an IM is to review study-specific training procedures. At an IM, ET s may be asked to:

- ✓ Deliver presentations and training packages in various modes, including large seminars or small group/individual trainings
- ✓ Provide Comprehensive COA training for CEs who have not received previous advanced level training

f. Data Analysis

The ET may assist the study sponsor in data analysis at the conclusion of trial and oversight of COAs for data lock.

10 Expert Trainer as an ongoing Support to site CEs

We recommend that a study research program include one lead ET who interfaces with the study sponsor and leads a training team to implement the training plan and QC around COAs. If the trial engages multiple sites, it is beneficial to have trainers located a similar geographical region to the CEs. The assigned ET will be the point of contact to a study site, serving as the focal point of support in study COAs. In addition, the ET will provide oversight on the qualification, training, and re-training of a CE in their geographical region and convey any concerns back to the lead ET and study management team. Via video, the ET will perform QC reviews to ensure quality clinical assessments are conducted by all CEs.

The ET should be available to provide support to site CEs by email, telephone, and/or in-person visits based on the needs of the site and sponsor.

11 Expert Trainer Role in Onsite Training

The ET may deliver onsite training in the event a CE is unable to attend the IM centralized training, or a new CE is required mid-study. Onsite training is to follow the same presentations and training packages as used in the IM for standardization purposes. Onsite training may also take place in the event of re-training or establishment of reliability.

The responsibility for training different outcomes and delivering various presentations is coordinated between the ET and the study sponsor to ensure all ETs have consistent training standards across the range of COA measures.

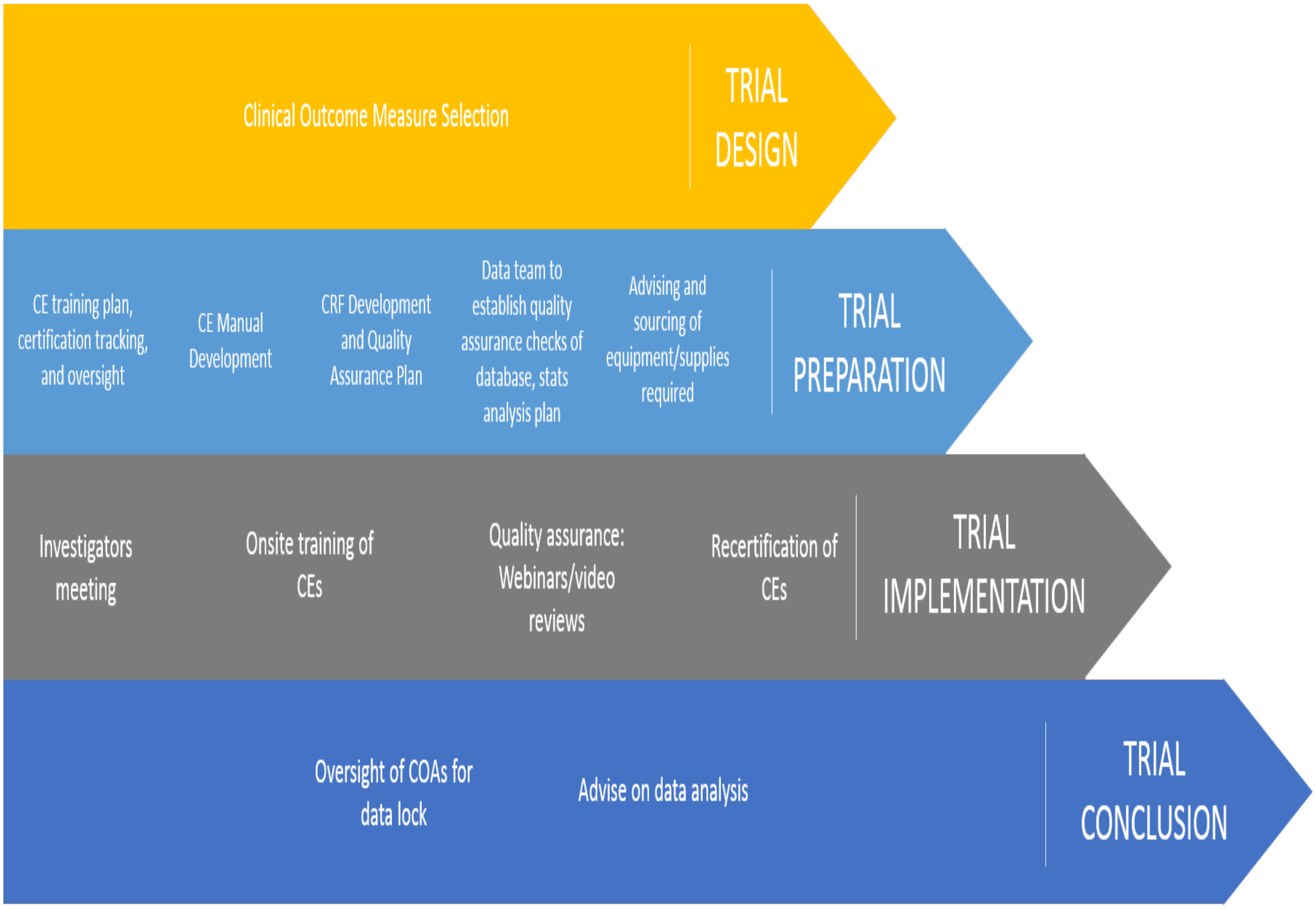

Figure c. *ET responsibilities at different stages of clinical trials*

12 Example of COA in DMD

Core COAs Utilized in DMD

Below is a list of COAs currently utilized in trials of DMD that will be covered during training and retraining. This toolbox is adapted from the World Health Organization Model and currently validated outcomes in DMD.

<table>
<tr><th colspan="2">International Classification of Functioning Disability and Health (ICF) Model</th></tr>
<tr><td>Impairment</td><td>Manual Muscle Testing (MMT)
Handheld Myometry (QMT)
Fixed System QMT
Range of Motion (ROM)
Pulmonary Function Testing</td></tr>
<tr><td colspan="2">Training will include use of equipment, standardization of positioning, test implementation.
Specific muscle or joint assessment will be protocol specific</td></tr>
<tr><td>Function</td><td>Northstar Ambulatory Assessment (NSAA)
Performance Upper Limb Scale (PUL2.0)
Egen Klassification Scale (EK)
Timed function tests (10meter, Supine to stand, 4 stairs)
Six-minute walk test
100-meter run
Reachable workspace</td></tr>
<tr><td colspan="2">Training will include standardization of testing positions and operational definitions of compensations
Training will follow published test validation</td></tr>
<tr><td>Quality of Life</td><td>PODCI
PEDSQL</td></tr>
</table>
